# Next-Generation Sequencing in Acute Lymphoblastic Leukemia

**DOI:** 10.3390/ijms20122929

**Published:** 2019-06-15

**Authors:** Nicoletta Coccaro, Luisa Anelli, Antonella Zagaria, Giorgina Specchia, Francesco Albano

**Affiliations:** Department of Emergency and Organ Transplantation (D.E.T.O.), Hematology Section, University of Bari, 70124 Bari, Italy; nicoletta.coccaro@uniba.it (N.C.); luisa.anelli@uniba.it (L.A.); antonella.zagaria@uniba.it (A.Z.); giorgina.specchia@uniba.it (G.S.)

**Keywords:** ALL, acute lymphoblastic leukemia, NGS, next-generation sequencing, third generation sequencing, MRD, minimal residual disease, targeted therapy, precision medicine

## Abstract

Acute lymphoblastic leukemia (ALL) is the most common childhood cancer and accounts for about a quarter of adult acute leukemias, and features different outcomes depending on the age of onset. Improvements in ALL genomic analysis achieved thanks to the implementation of next-generation sequencing (NGS) have led to the recent discovery of several novel molecular entities and to a deeper understanding of the existing ones. The purpose of our review is to report the most recent discoveries obtained by NGS studies for ALL diagnosis, risk stratification, and treatment planning. We also report the first efforts at NGS use for minimal residual disease (MRD) assessment, and early studies on the application of third generation sequencing in cancer research. Lastly, we consider the need for the integration of NGS analyses in clinical practice for genomic patients profiling from the personalized medicine perspective.

## 1. Introduction

Acute lymphoblastic leukemia (ALL) is the most common childhood tumor [1], showing 5-year survival rates of about 90% in children [2,3,4], and 75–85% in adolescents and young adults [3]. It accounts for about 15–25% of adult acute leukemias, and is characterized by different biological peculiarities as compared to the pediatric form, the outcomes in older adults being inferior and featuring overall survival rates of 35–55% in middle aged adults and under 30% in adults over age 60 [5,6,7,8]. ALL originates from the malignant transformation of B- and T-lineage lymphoid precursors and is triggered by a variety of genetic aberrations including chromosome translocations, mutations, and aneuploidies in genes responsible for cell cycle regulation and lymphoid cells development [9]. B-ALL and T-ALL subtypes show distinct patterns of genomic alterations and gene expression signatures [10]. Multiagent chemotherapy regimens are the current front-line therapeutic approach for both pediatric and adult ALL, followed by hematopoietic stem cell transplantation in high-risk groups [5,11,12,13]. New agents for ALL treatment are monoclonal antibodies, immunomodulators, and chimeric antigen receptor T cells (CAR-T); moreover, several new drugs targeting molecular pathways involved in leukemic cell proliferation have been identified [14]. Monoclonal antibodies such as Blinatumomab and Inotuzumab Ozogamicin (InO) were recently approved by the Food and Drug Administration (FDA) for the treatment of relapsed or refractory (R/R) adult ALL whereas CAR-T cells were approved for children and young adults with R/R ALL [14]. Molecular subtype classification greatly affects the treatment outcomes; over the past 60 years 5-year event-free survival has increased from less than 10% to around 90% in children thanks to new drugs development and adopting risk-adapted personalized therapy [15,16,17,18]. Despite this, relapse occurs in about 20% of pediatric and more than 50% of adult cases, together with a high rate of development of chemoresistance, treatment failure, and death [19,20,21,22]. Therefore, the establishment of diagnostic and/or prognostic criteria for risk classification to guide individualized clinical regimen determination and better predict the treatment outcome is of the utmost importance [10,15,18,23].

In this context, genetic analysis is the most powerful tool to identify the genomic alterations for the purposes of diagnosis, risk determination and treatment choice in ALL. However, two levels of genomic analysis can be recognized: the discovery phase, whose aim is to seek new molecular targets in order to gain a more precise understanding of the biology of ALL, and clinical practice, whose aim is to detect alterations that could contribute to the best management of the disease.

ALL genomic analysis in clinical practice is frequently conducted through the so-called “low-throughput techniques” (e.g., fluorescence in situ hybridization—FISH), which allow investigation of the most frequent abnormalities for ALL subtypes classification (*ETV6–RUNX1* fusion, *TFPT–PBX1* fusion, *BCR–ABL1* fusion; *MLL* fusions). Despite the limited number of detectable alterations, these are still largely used in most clinical laboratories worldwide. However, while awaiting modernization of the diagnostic and prognostic investigations, NGS technologies represent the future also in clinical practice, especially since the costs of these analyses are dropping fast and the knowhow and bioinformatics pipelines are increasingly easy to use. Today’s challenge is to understand how to integrate high tech molecular testing for patient profiling and stratification in daily clinical practice. The aim of our review is to consider the most updated discoveries obtained in the last four years during NGS studies for the diagnosis, risk stratification, treatment planning and response assessment in ALL, including the first studies about NGS use and implementation for minimal residual disease (MRD) guided approaches. We also review the first reports about the adoption of NGS in clinical practice and discuss the possibility and practicality of introducing the use of third-generation sequencers in this field.

## 2. ALL Genomic Analysis

Before the introduction of high-throughput sequencing, intensive genome-wide research was conducted using genomic and transcriptome microarrays. These studies led to the identification of important molecular targets and pathways associated with high-risk disease [1,24,25,26,27,28,29,30]. Genomic analyses revealed that ALL patients lack large genomic instability, even if tens of recurrent copy number alterations (CNAs) have been identified, involving genes serving for lymphoid growth or tumorigenesis [1,15,31], such as the B-lymphoid development regulators *PAX5* and *IKZF1*, tumor suppressors such as *CDKN2A*/*CDKN2B* and *RB1*, and drug response-related genes like *NR3C1* [32]. Frequently, these genes are affected by only one type of alteration such as focal or broad deletions, translocation, or mutations, acting in a haploinsufficient or dominant-negative manner [33]. Moreover, these works led to the identification of several alterations in T-ALL such as *LMO2* [34], *MYB* [35,36], and *PTEN* [37] and *WT1* [38]. 

Transcriptome microarrays studies led to the discovery of the *BCR-ABL1*-like or Philadelphia- like (Ph-like) ALLs, a new subtype of high risk B-ALL, whose expression shows signature clusters with *BCR-ABL1* positive cases [30,39]. These patients seem to respond less well to chemotherapy and have a higher risk of relapse than with other subtypes [30]; however, some of them respond to therapy with tyrosine kinase inhibitors (TKIs) (e.g., imatinib). Moreover, microarray analyses found alterations involving the *CRLF2* gene, demonstrating its overexpression in one third of *BCR-ABL1*-like cases and more than half of ALL patients with Down syndrome [26,40]. Frequently, this alteration appears concomitantly with gain-of-function mutations of *JAK1* or *JAK2* genes [26,40,41]. In the case of T-ALL, ETP T-ALL, a subtype similar to that of early thymic progenitors (ETP) and with a poor outcome, has been identified; it features an abnormal expression of CD1A, CD5, and CD8, and has distinctive gene expression signatures with some myeloid and stem cell traits [42,43]. 

While microarray approaches have largely contributed to extending our understanding of the biology of ALL, they present a series of limitations that penalize their application in the future, as it is not easy to recognize point mutations, chromosomal rearrangements, focal aberrations such as small insertions/deletions (INDEL), or structural variations (SVs). All these limitations have been exceeded by NGS technology, and this is why the most recent investigations prefer NGS use for a deep and comprehensive genome investigation.

## 3. Next-Generation Sequencing (NGS)

NGS refers to a series of modern massively parallel sequencing technologies. Based on the complexity of the analysis and the information to be obtained, several kinds of sequencing experiments can be performed, including whole genome sequencing (WGS), transcriptome sequencing (RNA-seq), whole exome sequencing (WES) and targeted gene sequencing. However, no single type of sequencing is capable of detecting the same alterations. So, WES is useful for point mutation investigation, particularly on leukemic subclones during relapse and is therefore usually executed with high coverage [44]; whereas WGS can reveal SVs. RNA-seq is used to analyse the expression of mRNA or noncoding RNA, and can identify sequence mutations as well as fusion genes [45], which can also be detected by WGS.

A classical bioinformatic workflow for WGS, WES and targeted gene sequencing data analysis comprises three main phases: ‘alignment’, ‘variant calling’, and ‘filtering and annotation’. In the alignment step, each of the short reads generated from the NGS experiment is matched to positions on the human reference genome, resulting in a sequence alignment file stored as a sequence alignment/map (SAM) or binary alignment/map (BAM) file. Then, there is the variant calling in which the aligned sequences are compared with reference sequences to find the positions that deviate from the reference, producing a list of calls detailed in a variant call format (VCF) file. The last step consists of variant filtering and annotation: through filtering, the produced variants are reduced to a smaller set; generally, variants located in genomic duplicated regions or present in the 1000 genome project or the Exome Aggregation Consortium (ExAC Version 0.3) at a frequency higher than 1% are discarded [46]. Through annotation known information about each detected variant is queried in order to try to explain its biological effect. 

Each phase of this computational framework can be conducted with a plethora of standard software, some of which are free and others commercially available; for more specialized tasks customized scripts often need to be written and adapted pipelines are adopted. For example, the reads resulting from WGS experiments can be mapped to the human genome with different alignment software, for example the Burrows–Wheeler Aligner MEM (version 0.7.12) pipeline [46,47]. Also, in WGS experiments somatic mutations (single nucleotide variants and short INDEL) can be identified by using different callers such as MuTect25 [46,48], GATK9 [48], Bowtie 2 [47], Strelka54 [49]. For annotation, several programs can be applied such as ANNOVAR, that consults varies annotation databases such as RefSeq12, CADD13, SIFT14 and PolyPhen215 [46].

Similarly, for RNA-seq data experiments several spliced aligners are available; for example, TopHat aligns RNA-Seq reads using the read aligner Bowtie, and analyses the mapping results to identify splice junctions; its last version, TopHat2, can also align reads across fusion breaks, which can occur after genomic translocations, and thus it has been employed for the search of fusion genes in ALL [46,49,50]. Other similar less-used software have been used for similar purposes, such as FusionCatcher, Cicero, Chimerascan 49 (0.4.5) and Defuse [46,48,49,50,51]. For differential expression analysis, relative transcript expression levels can be calculated based on supporting reads for each gene determining the ‘Fragments Per Kilobase of transcript per Million mapped reads’ (FPKM). The read counts can be obtained with different bioinformatic pipelines, for example the HTseq-count program [48,50,51,52], and the resulting FPKM values are then analysed by algorithms such as Cufflinks 2.2.0 [46,49], DESeq2 package [48,50] or Qlucore Omics Explorer (v3.1) [49]. 

The resulting alignment and VCF files can be visually inspected with several tools; one of the most used is the Integrative Genomics Viewer (IGV) software. 

Since the first experiments were performed in 2001, the costs for a WGS have drastically lowered from one hundred million to one thousand USD, and the price is expected to fall further. In parallel the bioinformatics tools used to handle big sequencing data have been enhanced thanks to the development of new algorithms and pipelines, rendering the data analysis phase easier. So, over time these approaches have become increasingly accessible both from the economic point of view and from the point of view of ease of use; accordingly, nowadays most laboratories around the world are being equipped with NGS. This is the reason why the number of studies involving large cohorts of patients has greatly increased in recent years and led to the discovery of new targets and new molecular entities. Several updates have been made about the first applications of NGS in the study of ALL [16,53]. Herein, we review the very latest works in this field from 2015 until today (Table 1).

### 3.1. New Insights into B-ALL Biology

The generation of fusion genes or the altered expression of key genes in lymphoid development are among the most frequent aberrations found in ALL [54]. In the last few years, the application of NGS in large genomic studies has contributed to extending the number of known molecular entities and to expanding the information about those already known.

#### 3.1.1. *DUX4/ERG* ALL

Through an integrated approach comprising NGS and array in 2016, four independent studies identified a new subclass of B-ALL characterized by the alteration of the homeobox transcription factor gene *DUX4* [47,48,49,52]. The *DUX4* aberration accounts for 4–7% of B-ALL and features a rearrangement and overexpression of *DUX4* and transcriptional deregulation or deletion of the transcription factor gene *ERG* [49,52]. In some cases, there is also the expression of *ERG_alt_*, a novel *ERG* isoform generated by the use of a non-canonical first exon whose transcription was initiated by DUX4 binding [52]. It acts as a dominant negative inhibitor of wild type *ERG*, resulting in loss-of-function of *ERG* [52]. Aside from the *ERG* deletions, *DUX4* fusions are frequently accompanied by *IKZF1*, *PAX5* and *CDKN2A*/*CDKN2B* deletions, and by activating mutations in *NRAS* and *KRAS* [48,52] as well as *MYC*, *MYCBP2*, *MGA*, and *ZEB2* [52]. In the study by Yasuda et al. RNA-seq was applied to analyse a cohort of 73 adolescent and young adults ALL cases (AYA-ALL; 15–39 years old) and frequent insertion of *D4Z4* repeats containing the *DUX4* gene in the *IGH* locus was found, causing overexpression of DUX4 protein with an aberrant C terminus [47]. Subsequent in vivo studies in mice inducing the expression of *DUX4*-*IGH* in pro-B cells showed the development of leukaemia. The preferential recurrence of the *DUX4* fusions in AYA-ALL led the authors to conclude that these aberrations could characterize a distinct clinical entity from ALL at other ages [47].

#### 3.1.2. *ETV6-RUNX1*-Like ALL 

Another novel ALL subclass defined as ‘the *ETV6-RUNX1*-like’ was identified in the same studies that demonstrated *DUX4* aberrations [48,49]. This new category is characterized by *ETV6* and *IKZF1* aberrations and by the same gene-expression profile as *ETV6-RUNX1* positive leukaemia [48,49]. This ALL subclass was identified also in two other recent reports by Zaliova et al. [55,56]. All the *ETV6-RUNX1*-like cases presented *ETV6* alterations and a CD27pos/CD44low-neg immunophenotype, typical of *ETV6-RUNX1*–positive patients [55,57], as well as *IKZF1* and *ARPP21* aberrations [49]. Together, these researches reveal that about 3–5% of all childhood BCP-ALL cases belonged to this new biological category, extending the list of ALL-associated fusion genes and adding new elements to improve risk stratification, even if studies on larger cohorts are needed to clarify the prognostic value of this new subclass.

#### 3.1.3. MEF2D/ZNF384 ALL

The work by Liu et al. applied WES, RNA-seq and SNP arrays to investigate the genomic composition of 203 B-ALL cases (92 adult and 111 paediatric), and of 87 other adult and 93 paediatric cases to seek for recurrent gene mutations and fusions [48]. These analyses allowed them to recognize eight gene expression subclasses with a strong correlation with some gene fusions, karyotype alterations or intra-genic deletions, besides demonstrating an association with certain immunophenotypes. A subsequent large-scale study, including 1,223 BCP ALL cases analysed by RNA-seq, extended the eight expression subgroups identified by Liu et al. 2016 to 14, adding six further subgroups [58]. Noteworthy, *MEF2D* and *ZNF384* fusions were found as recurring events both in adult (about 7%) and in paediatric cases (about 3.6%) and characterized two distinct expression subgroups [48]. The expression subgroup characterized by *MEF2D* fusions showed the upregulation of *HDAC9* [59] and *PTPRZ1* that shape the B-cell repertoire [60]. Subsequent functional in vivo and in vitro analyses demonstrated that such fusions could damage B-cell development; *MEF2D* fusions altered *HDAC9* expression, in turn acting on the repression of B-lineage genes (*RAG1*) [48]. *MEF2D*-rearrangements were also identified in another parallel study with RNA-seq by Gu et al. in a discovery cohort of 560 leukemic patients and subsequently in an extended cohort of 1,164 B-ALL cases. They revealed a high-risk B-ALL subgroup with poor prognosis both in adult and in paediatric cases [50], as in the study by Liu 2016. They found, in *MEF2D*-rearranged cases, a distinct immunophenotype featuring CNAs at the rearrangement sites, older age at diagnosis and poor outcome. Indeed, an enhanced *MEF2D* transcriptional activity was observed, leading to the activation of *HDAC9* expression and sensitivity to histone deacetylase inhibitor therapy. This observation suggested a new treatment approach for this ALL subtype [48,50].

The expression subgroup characterized by *ZNF384* fusions exhibited few aberrations of genes involved in B-cell development (e.g., *IKZF1*, *PAX5*, *RUNX1*, *ETV6*) or the cell cycle (e.g., *CDKN2A/2B*) and a hyper-expression of *JAK-STAT* signalling pathway genes and of *GATA3* [61], *CEBPA* and *CEBPB* [62], all transcription factors fundamental in the reprogramming of B-cells to myeloid cells [48]. As regards *ZNF384*-fusions, whole-transcriptome sequencing analysis of 231 childhood ALL cases found 58 fusion genes in 125 patients (54.1%), 31 of them never described before [63]. In addition, a separate ALL subclass was identified in the rearrangements of the *ZNF384* gene with EP300 and CREBBP, two histone acetyl transferases, characterized by a specific expression signature, with overexpression of *CLCF1* and *BTLA* [63]. In vitro and in vivo assays on the *EP300-ZNF384* and *CREBBP-ZNF384* fusions demonstrated differentiation alterations of hematopoietic stem and progenitor cells stimulating leukemic conversion, maybe through a global epigenetic alteration caused by the reduction of histone acetylation with a fusion protein acting in a dominant-negative manner on the wild type counterpart of histone lysine acetyltransferase [48,63]. This evidence suggested that leukemic cells are sensitive to histone deacetylase inhibitors, opening up new treatment perspectives for this class of patients [63]. Indeed, as *EP300-ZNF384*-positive patients showed up-regulation of JAK-STAT pathway, it was also suggested that they could be treated with JAK-STAT inhibitors [48].

#### 3.1.4. *TCF3-HLF* ALL 

In the attempt to investigate the biologic landscape of *TCF3-HLF*−positive ALL, characterized by a very poor prognosis, Fischer et al. adopted an integrated NGS approach [64]. They found an association of *TCF3-HLF*−fusion with *PAX5* haploinsufficiency and enrichment in stem cell and myeloid expression signatures; these alterations could reprogram the cell toward an immature drug-resistant hematopoietic state. New drug response profiling found sensitivity to glucocorticoids, anthracyclines, and especially toward the BCL2-specific inhibitor venetoclax (ABT-199), suggesting new treatment alternatives for this lethal ALL subtype [64].

#### 3.1.5. *MLL*-Rearranged (*MLL*-R) ALL

ALL with Mixed Lineage Leukaemia (*MLL*) gene rearrangements is an aggressive form of leukaemia. In order to explore the genetic bases of the *MLL*-rearranged (*MLL*-R) ALL, in 2015 Andersson et al. conducted WGS, WES, RNA-seq and targeted sequencing of 67 *MLL*-R cases (47 *MLL*-R infants and 20 older children) and 18 non–*MLL*-R cases [65]. Intriguingly, infant *MLL*-R leukaemia cases showed a very low frequency of somatic mutations (a mean of 1.3 non-silent mutations), maybe because of the extreme oncogenic power of the MLL chimeric proteins. Indeed, the few observed mutations struck kinase-PI3K-RAS signalling components in 47% of cases, corroborating the interplay between *MLL*-fusions and that pathway [66,67,68]. Instead, older *MLL*-R children presented a higher frequency of somatic mutations in epigenetic regulators (45%); however, these mutations were frequently recovered as subclones and lost at relapse [65]. This observation led to the concept that these variants do not concur in the generation of the dominant leukemic clone and that maybe targeting them with specific substances will not improve the therapeutic response. Thus, *MLL*-fusions or other components required for its biological function must remain the main target of the therapy against this kind of ALL [65].

#### 3.1.6. ALL Negative for the Known Fusion Transcripts 

WES and targeted sequencing were performed in 168 cases including children, AYA and adults to investigate the pathogenic landscape of the so-called B-NEG ALL, i.e., ALL cases negative for the known fusion transcripts, such as *BCR-ABL1*, *ETV6-RUNX1*, *E2A-PBX1*, and *MLL* rearrangements [69]. A rate of 10.5 mutations and 9.1 CNAs/sample was found, RAS/RTK (26.8%) and JAK/STAT (12.5%) signalling being the most mutated and druggable pathways. In paediatric cases *KRAS* and *NRAS* were more frequently altered, whereas in AYA and adult cases *FLT3* and *JAK/STAT* mutations were mainly identified. *RAS/RTK* mutations and *JAK/STAT* mutations impaired the outcome of adults, but not of paediatric cases; *RAS/RTK* alterations affected the prognosis of AYA. In vitro studies found *FLT3*-mutated cells sensitive to FLT3 inhibitors, and *RAS* and *IL7R* mutated primary cells sensitive to PI3K/mTOR inhibitors, thus demonstrating that these inhibitors might be incorporated in the treatment of ALL [69].

#### 3.1.7. *EPOR* Gene Rearrangements

A subclass of Ph-like ALL is characterized by chromosomal rearrangements involving the erythropoietin receptor gene, *EPOR*. In 2016, in a study by Iacobucci et al., the incidence of EPOR rearrangements was analysed by WGS and RNA-seq in 3115 childhood, adolescent and young adult B-ALL patients, including 212 cases with a Ph-like ALL gene expression profile. Only 19 cases were identified among the Ph-like patients; all of them presented the truncation of the cytoplasmic tail of EPOR, causing alterations of EPOR expression, an increased sensitivity to erythropoietin, and JAK-STAT pathway hyperactivation [70]. The altered amino acid positions were similar to those involved in primary familial congenital polycythemia, with loss of distal regulatory residues and conservation of the proximal tyrosine essential for receptor activation. Indeed, frequently such rearrangements were accompanied by aberrations in the *CDKN2A*/*CDKN2B*, *IKZF1* and *PAX5* genes. As in vitro studies on leukemic cells with *EPOR* rearrangements responded to JAK-STAT signalling inhibitors, the authors suggested this as a new treatment possibility for this patients category [70].

#### 3.1.8. Aneuploid ALL

Aneuploidy is one of the hallmarks of ALL and NGS studies have been applied to explore the pathogenic causes responsible for its generation. About one third of infant BCP ALL presents high hyperdiploidy with more than 50 chromosomes. To understand the pathogenesis of high hyperdiploidy ALL, WGS and/or WES, of 16 and 39 diagnostic and remission samples, respectively, were performed [71]. In the majority of patients, the involvement of the RTK-RAS pathway and of histone modifiers was demonstrated, with no observation of recurrent fusion genes. Indeed, analysis of the mutations on trisomic chromosomes revealed that the chromosomal gains were early events, prompting the hypothesis that the high hyperdiploid condition is the main leukemic driver event [71]. Compared to hyperdiploidy, hypodiploidy with less than 44 chromosomes is less frequent, being observed in about 3% of ALL patients. A strategy involving WES, RNA-seq, SNP array and methylation array was adopted to study 11 hypodiploid (<45 chromosomes) ALL cases [72]. The high hypodiploid ALL class (40–44 chromosomes) showed gross chromosomal instability, displaying single variability in chromosomal content. As regards mutations, *IKZF3* and *FLT3* mutations were observed in near-haploid (25–30 chromosomes) patients, as previously reported [73]; low hypodiploidy (31–39 chromosomes) cases displayed *TP53* mutations, that were also present in 3/3 high hypodiploidy cases, suggesting that the mutational patterns are similar in low hypodiploid and high hypodiploid ALL. In hypodiploid ALL, gene expression analysis found an overexpression of genes on heterodisomic chromosomes. When the cases were grouped according to the hypodiploid state no association between chromosomal copy number and methylation levels was found. In three quarters of cases, relapse did not arise from the clone responsible for the onset. The authors concluded that the mutational landscape of near-haploid and low hypodiploid ALL is different [72].

#### 3.1.9. Inherited ALL

An inherited predisposition to ALL is rare; however, some inherited variants have been demonstrated to increase the risk of susceptibility to ALL [74,75,76,77,78,79,80,81,82,83,84], and several familial ALL cases were characterized by inherited deleterious mutations [73,85,86] in key genes as such as *TP53*, *RAS* signalling components [73], and *PAX5* [85,87]. In 2015, a novel genetic syndrome associated with a predisposition to childhood ALL was identified through WES analysis of an index family with several cases of ALL in which a new germline non-sense ETV6 variant (p.Arg359X) was found [87]. In a following targeted sequencing of *ETV6* in 4405 childhood ALL patients, thirty-one exonic variants possibly linked to ALL risk were found in 1% of cases (35 patients) [88].

### 3.2. New Insights into T-ALL Biology

NGS studies were performed also in T-ALL cases, that account for about 15–25% of ALL and are sub classified according to stages of thymic maturation: early cortical, late cortical and mature T-cell stages. 

#### 3.2.1. Coding and Non-Coding Gene Mutations 

Three independent groups performed targeted sequencing of genes known to be altered in T-ALL [89,90,91]. Besides the well-known mutated genes *NOTCH1*, *FBXW7*, *WT1*, *JAK3*, *PHF6*, and BCL11B, whose mutation frequencies were consistent with literature data, they found aberrations of new transcription factors such as *DNM2* and *RELN*, *FAT1* (a cadherin associated with the WNT pathway) and of epigenetic regulators such as *MLL2* and *EZH2*. Moreover, new mutations were evidenced in *HERC1* (a DNA complex member), in *NOTCH2*, and in the splicing factor *ZRSR2* [90]. Overall, the JAK/STAT pathway and epigenetic regulation members were altered in 18% and 33% of cases, respectively, revealing an association with the unfavourable subgroup of early T-ALL [90,91]. Noteworthy is the fact that some mutations were revealed as subclones, responsible for treatment resistance and progression of relapse [90]. However, the involvement of such pathways suggested the potential clinical use of JAK inhibitors to treat such ALL cases [89,90]. New gene variants were identified by Chen et al., in a 2018 WES and RNA–Seq study conducted in 130 T-ALL cases (61 adult and 69 paediatric) [92]. They found 48 genes with mutation rates >3% (among which six never described mutations in *PAK4*, *CELSR3*, *MINK1*, *NR4A1*, *BOD1L1*, and *VCP*) and clustered them in seven functional categories: NOTCH1 pathway, signalling pathways, epigenetic factors, TFs, cell-cycle regulators, translation and RNA stability-associated molecules, as well as others. Almost all cases with gene fusions, which were mutually exclusive, presented the concomitant mutation of a gene (95.7%), even if *FBXW7* and *DNMT3A* mutations were found more frequently in cases without fusions. Notably adult ALL presented higher mutation frequencies than paediatric cases, especially among signalling pathways and epigenetic factors categories. Indeed, there was a significant correlation between age and the number of gene mutations. Furthermore, *CDKN2A* and *CDKN2B* deletions were identified in 75 and 64 of 115 cases, respectively.

Functional non-coding genomic aberrations in T-ALL cases were specifically investigated by Hu et al. 2017 with WGS and RNA-seq analyses [46]. Besides the well-known coding mutations in *NOTCH1*, *FBXW7*, *USP7*, and *PTEN*, most variants mapped in non-coding regions. After filtering, the most relevant enrichment for non-coding mutations involved the *LMO1*, *LMO2*, and *TAL1* loci, that code for transcription regulators strictly regulated during thymocyte differentiation [93,94,95,96,97,98], whose overexpression has been described in T-ALL patients. The observed alterations provoked transcription deregulation, and, in the case of the *LMO1* locus, displayed remarkable positional conservation (being located in binding motifs for specific T-ALL transcription regulators such as *MYB*, *CREBBP*, and *RUNX1*), suggesting a selection pressure likely in favour of the aberrations triggering the most relevant overexpression of *LMO1*. Also in the case of the *TAL1* enhancer mutation the same mechanism was observed; for *LMO2* intronic mutations, instead, there was no clear significance, as they were not significantly associated with *LMO2* expression alteration, even if the authors hypothesized *MYB* involvement [46].

A large cohort of childhood and young adult T-ALL cases was investigated by Liu et al., 2017 [51] with an integrated genomic approach. Although the most frequent T-ALL mutations had already been reported, gene variants were identified in 106 putative driver genes, half of which never described in childhood T-ALL (e.g., *CCND3*, *CTCF*, *MYB*, *SMARCA4*, *ZFP36L2* and *MYCN*). Several gene mutations were identified in association with specific stages, subtypes, cellular pathways and outcome. For example, *NRAS*/*FLT3* mutations were more frequently found in immature T-ALL, *JAK3*/*STAT5B*, *PTPN2*, and *PIK3R1*/*PTEN* mutations were associated with *HOXA1*, *TLX1*, and *TAL1* deregulated ALL, respectively. 

#### 3.2.2. New Fusion Genes and Gene Expression Deregulation

In the previously cited study by Chen et al., 2018, 36 fusion genes were revealed, among which 18 were the first observed, with *ZBTB16*-*ABL1*, *TRA*-*SALL2*, and *NKX2-1* rearrangements as recurrent events [92]. *ZBTB16* is a hematopoietic regulator and is known to be a fusion partner of the retinoic acid receptor alpha (*RARA*) gene in a subgroup of acute promyelocytic leukaemia (APL) cases [99]. In the *ZBTB16-ABL1* fusion protein found in the work by Chen et al., 2018, *ZBTB16* retained the same portion as in *ZBTB16-RARA*, and ABL1 conserved the SH3, SH2 and the tyrosine kinase domains, preserving PTK activity which could confer a strong transforming power [100]. In mice, in vivo studies showed a response to PTK inhibitors, prompting their use in *ZBTB16-ABL1*-positive cases. Through unsupervised clustering methods, three distinct expression subgroups were identified with a predominant *TLX1*/*TLX3* overexpression, *LYL1* expression, and *TAL1* and *LMO1* overexpression. The search for abnormal mRNA splicing products or transcripts found the aberrant overexpression of the short mRNA transcript of the *SLC17A9* gene in most cases with TAL1 overexpression, associated with a poor prognosis in the adult cohort. *TAL1* up-regulation characterized the paediatric cohort, instead *HOXA*, *MEF2C*, and *LYL1* overexpression was mostly found in adults. The authors highlighted the concomitant overexpression of *SPI1* and *MEF2C* in a subset of cases and observed that the former acts as a regulator for the latter in normal lymphoid development, suggesting that dysregulation of this pathway could be a driver for leukemogenesis [92].

#### 3.2.3. ETP-ALL

ETP-ALL is a recently described ALL subtype with poor prognosis accounting for 5–16% of T-ALL, characterized by a unique immunophenotype, with a reduced expression of several T cell antigens, and aberrant expression of myeloid and stem cell markers [42]. ETP-ALL is genetically heterogeneous but features a high frequency of mutations in myeloid genes such as *FLT3* and *NRAS* [101]. However, disease specific mutations have not yet been identified. WES analysis of five paired ETP-ALL samples was performed by Neumann et al., 2013 [102]. In addition to known mutations in genes such as *ETV6*, *NOTCH1*, *JAK1*, and *NF1*, novel recurrent variants were revealed in *FAT1* (25%), *FAT3* (20%), *DNM2* (35%), and in epigenetic regulators such as *MLL2*, *BMI1*, and *DNMT3A*. A frequency of about 16% of *DNMT3A* mutations was identified in a larger cohort of adult ETP-ALL patients (10/68). Overall, this study showed that more than 60% of adult ETP-ALL patients are characterized by a least a single mutation in *DNMT3A*, *FLT3*, or *NOTCH1* that could guide therapeutic choices in this high-risk subgroup. A more recent WGS analysis study was performed on an adult ETP-ALL case to identify clonal and sub-clonal mutations for subsequent monitoring of MRD [103]. As disease-specific mutations have not yet been identified in ETP-ALL, twelve somatic mutations in known oncogenes were selected and analysed by NGS at distinct time points during the follow-up; this study revealed that WGS analysis for identification of multiple target genes is a precise method for subclonality analysis and for the identification of useful targets in MRD monitoring [103]. 

#### 3.2.4. Relapsed T-ALL

To explore the pathogenic mechanisms of relapse and to detect the occurrence of *NOTCH1*/*FBXW7* alterations in relapsed T-ALL, a WES study was conducted in 30 paediatric patients, among which 11 diagnosis-relapse paired cases [104] *NOTCH1*/*FBXW7* mutations were identified in 73.3% of cases at the onset and 72.7% of the relapses. At diagnosis, mutations in the heterodimerization domain predominated (40.0%), while at relapse PEST domain alterations were more frequent (54.5%). In the diagnosis-relapse paired cases, there was a prevalence of PEST mutations in relapsed cases, although this observation was not confirmed in the target cohort. Indeed, in two of 11 diagnosis-relapse paired cases a mutation switch was observed from diagnosis to relapse [104] Despite the small sample size, these data suggested that the presence of *NOTCH1* mutations might contribute to the T-ALL relapse. 

### 3.3. MRD Monitoring by High Throughput NGS (HT-NGS)

MRD monitoring is currently performed in all paediatric ALL and in a large number of adult patients to evaluate treatment response and to define MRD based risk stratification [6,105,106]. Patients with good prognosis show undetectable MRD levels after induction therapy, whereas persistent MRD defines high relapse-risk patients [107,108]. At present, classical techniques for MRD monitoring are flowcytometry and polymerase chain reaction (PCR) applications. MRD analysis needs to be accurate and sensitive (≤0.01% or ≤10^−4^) and requires highly specific markers for ALL cells, such as abnormal immunophenotypes, specific molecular aberrations (fusion genes or point mutations), and immunoglobulin (*IG*) or T-cell receptor (*TCR*) gene rearrangements. However, classical flow cytometry does not always reach a sensitivity of 10^−4^ [109] and quantitative PCR (RQ-PCR)-based on allele-specific oligonucleotide (ASO) assays for the detection of *IG* and *TCR* rearrangements is a time-consuming and laborious experimental procedure [110]. A recent work showed that another useful and robust method for monitoring MRD in adult Ph-positive ALL is droplet digital PCR [111]; however, specific molecular markers such as the *BCR-ABL1* fusion gene are only available in a limited fraction of ALL patients. In the last few years, new highly sensitive technologies have been developed for MRD monitoring, consisting of > 8-color next-generation flow and in high-throughput NGS of *IG/TCR* rearrangements (*IG/TCR* HT-NGS) [110]. Nowadays, this last technique offers a crucial NGS application for almost all patients with lymphoid malignancies, avoiding the laborious design of patient-specific probes performed in ASO RQ-PCR assays. For this purpose, a multiplex PCR is carried out with a combination of V, D, and J universal primer sets to identify any possible *IG* rearrangement and to sequence small genomic regions with a sensitivity of 10^−4^–10^−6^ [110,112]. The higher sensitivity could allow MRD monitoring from peripheral blood avoiding painful bone marrow aspirations [113,114]. Moreover, because of its high depth, *IG/TCR* HT-NGS obtains sequencing information from each molecule separately and reveals many more gene rearrangements than classical methods; therefore, it is crucial to define which of the multiple identified rearrangements are most common and should be selected for MRD monitoring [115]. In fact, based on NGS analysis, most ALL patients were revealed to be oligoclonal, with a median of about 20 clonal rearrangements per case; however, in the majority of patients the most frequent clone at diagnosis will still be present at relapse, although clonal evolution with new emerging clones is a common phenomenon [116,117]. A previous study comparing diagnostic and relapse samples of paediatric ALL by WGS and WES analyses identified several mutated genes associated with relapse such as *NRAS*, *KRAS*, and *PTPN11*; the clonal evolution and the selection of these mutations seemed to be chemotherapy-driven [118]. Moreover, the turnaround time for the *IG/TCR* HT-NGS analysis is about 1 week per sample and MRD quantification could be performed on day 15, an early treatment time point that is a powerful relapse predictor in paediatric ALL [119]. Several studies have compared the conventional RQ-PCR method to *IG/TCR* HT-NGS, demonstrating a good correlation, excellent concordance and higher sensitivity with NGS than ASO RQ-PCR, without the need to identify patient-specific probes [120,121]. Another study revealed higher specific molecular MRD quantification of *IG/TCR* HT-NGS in two cohorts of paediatric ALL patients after SCT, that showed low levels of positivity with ASO RQ-PCR [122]. Moreover, several studies demonstrated that *IG/TCR* HT-NGS is a more sensitive method and allowed a better risk stratification of ALL patients on the basis of MRD levels, improving the distinction between deeply negative and very low positive cases and enabling an earlier identification of molecular relapse [113,121,123]. The conventional RQ-PCR method for MRD detection in ALL has been widely standardized within the EuroMRD consortium (http://www.euromrd.org/), that includes several laboratories around the world, organizes periodic quality control rounds and develops guidelines for MRD monitoring [124]. By contrast, HT-NGS for *IG/TCR* is a relatively new procedure and standardization is still lacking, therefore it is not yet a routine method for MRD monitoring [110,125]. Recently, FDA approved the first NGS assay (ClonoSEQ) for detecting MRD in patients with ALL or multiple myeloma [126]. Tests are performed on bone marrow samples to monitor of measurable residual disease at specific time points throughout a patient’s treatment. The ClonoSEQ Assay combines multiplex PCR and NGS to identify and quantify rearranged *IgH* (*VDJ*), *IgK*, and *IgL* receptor gene sequences in ALL patients, monitoring changes in disease burden during treatment, in conjunction with clinical guidelinREVISIONes. 

As the MRD monitoring method is based on fusion-genes identification, recent studies developed targeted NGS assays to detect *ETV6-RUNX1* and *MLL* gene rearrangements, demonstrating that the NGS methodology is more sensitive compared with current diagnostic methods such as inverse PCR and long distance PCR performed on RNA or genomic DNA [127,128]. NGS for identifying *ETV6-RUNX1* fusion allows the identification of new variant rearrangements that seem to be correlated to a worse prognosis, needs lower amounts of genomic DNA for the analysis, and is a simple and fast method that allows the processing of many samples simultaneously [127]. A broad range of possible *MLL* fusions was tested by a multiplex PCR method on RNA followed by a targeted NGS analysis, revealing a good concordance with the gold standard technique based on long-distance inverse PCR [128].

## 4. Third-Generation Sequencing

A special mention should be made of so-called ‘third generation sequencing’, also known as long-read sequencing, that allows the reading of sequences at the single molecule level and differs from second-generation technologies (i.e., NGS) based on amplification and synthesis of small DNA segments. MinION is a single molecule third-generation sequencer based on Oxford nanopore technology; it is a small size device connected to a laptop that operates by consecutively sequencing two strands of DNA molecules connected by a hairpin [129]. The single strand of DNA walks across a chip with biologic nanopores; by applying an electric field, the electrical signal variations in length generated along the way out of consecutive 5-bases DNA are recorded. Besides the dimensions, the main advantage is the generation of long reads up to about 1Mb, rendering it ideal for the study of large SVs, unlike traditional sequencing technologies based on short-reads. Indeed, it can perform direct acid nucleic sequencing both of DNA and RNA, and the cost of each analysis is very low. 

In the last three years, multiple publications have illustrated its wide range of applications in cancer research. For example, recently researchers from The Jackson Laboratory developed a customized pipeline, called Picky analysis, to elaborate data on the long reads of the nanopore platform for comprehensive detection of SVs in a breast cancer genome [130]. Indeed, a Nanopore-based sequencing assay has been developed for rapid and targeted identification of fusion oncogenes, that enabled a real-time analysis within just 5 min, with the first fusion read being generated within five seconds even with a low tumour burden [131]. Moreover, our group recently employed nanopore sequencing associated with long-range template multiplex PCR to analyse *BCR-ABL1* DNA fusions, comparing it to FISH followed by Sanger sequencing (SS), and we confirmed the advantages of MinION use as regards the very low costs, the ease of use, and the length of the reads [132].

Another convenient use of MinION is the detection and analysis of somatic mutations [133,134,135,136,137]. This method has already been successfully exploited to search for *TP53* gene mutations [133], and very recently, it has been applied to create a single assay to detect *FLT3* duplications [138]. By creating an RNA amplicon sequencing test covering the well-defined hotspot regions of *FLT3*, the nanopore approach offered rapid gaining of full-length reads, providing the recognition of mutations [138]. Furthermore, a customized gene panel has been created by our researcher team to analyse *TP53*, *NOTCH1*, *BIRC3*, *SF3B1* and *MYD88*, five recurrently mutated genes in chronic lymphocytic leukaemia [139]. In this work we demonstrated that nanopore sequencing is capable of successfully detecting somatic variants, with a final breadth of coverage of the panel of 94.1%, even if it is necessary to improve the accuracy of the analysis because of the error rate of about 6% for INDEL and 2% for single nucleotide variants [139]. In the field of the research on ALL, the first application of this third-generation sequencing technology was in the detection of *BCR-ABL1* KD mutations in Ph-positive leukaemia patients [134]. The onset of such mutations is the reason for treatment failure with TKIs in 25–50% of non-responding chronic myeloid leukaemia patients [140], and their frequency is much higher in patients with Ph-positive ALL at the time of relapse [141]. In our work, nanopore sequencing was compared to classic SS, revealing that MinION analysis was much better than SS as regards sensitivity, costs and duration. Indeed, it allowed the investigation of clonality of compound mutations [134]. 

All these studies demonstrated that nanopore sequencing looks like a promising forefront platform that will be useful in time-critical situations or for analysis of cases with a low specimen volume. Surely, studies on its applicability in clinical practice and on ALL genomics analysis are still in their infancy and further studies will be required to confirm its reliability and accuracy. Indeed, the main MinION challenge to be dealt with is the high error rate especially in homopolymeric sequences, for which basecalling is a crucial step; thus, several basecallers are being developed, trying to improve read accuracy [142], as well as new bioinformatic pipelines for variant calling analysis [137,143]. Indeed, studies of other single-molecule sequencing technologies are ongoing, attempting to defeat the MinION error rate [144,145]. In the future the integration of these advanced technologies in nanopore sequencing will likely yield a true technologic improvement that will bring advanced genomic analysis within everyone’s reach.

## 5. NGS in Clinical Practice

Nowadays, the physician is at a crossroads confronted on the one hand by the enormous amount of information generated by large-scale NGS studies, and on the other by the question as to how to apply this information in everyday clinical practice. While continuous discoveries enrich and expand our understanding of cancer at the molecular level, a practical way must be found to exploit them for the benefit of patients, to improve risk stratification, disease monitoring and new drug discoveries in the perspective of precision medicine. 

The present risk stratification and treatment schedules on ALL include age, sex, white blood cell count at presentation, central nervous system involvement, cytogenetic aberrations defined, and levels of MRD burden, so the incorporation of new genomic discoveries needs to be closely assessed. Indeed, we are facing a dichotomy between technologies used in discovery steps for aberrant genomic alterations, and cheaper faster methods adopted in clinical practice [146]. However, the clinical benefits of such implementation are clear, allowing a more careful patient management as regards diagnosis, prognosis, treatment choice, MRD evaluation and new drug discovery.

In 2017, the St. Jude Children’s Research Hospital group reported its experience in the implementation of NGS approach in the management of patients with ALL [147]. Beside the well-established morphologic, immunophenotyping, and molecular genetic approaches, for all consenting patients they also performed NGS diagnostics. All new diagnoses underwent RNA-Seq for fusion detection, allowing the identification of known and novel fusion transcripts, available by day 15. They reported the advantages of this approach, especially for identifying therapeutic targets for personalized precision medicine, reducing toxicities, especially in higher risk patients [147]. 

Surely the implementation of NGS in clinical practice for MRD monitoring is one of the most important next goals, bearing in mind the promising improvements as regards sensitivity and ease of application compared to the older standard methods, as previously discussed in Section 3.3.

## 6. Conclusions

In the NGS era, previous techniques have been replaced by more efficient methods of genome-wide analysis. Now, the use of NGS technologies has led to advances in the knowledge regarding tumour genomic heterogeneity, with substantial implications on the selection of specific molecular biomarkers and clinical decision making for precise therapeutic approaches. Treatment response varies based on each peculiar genomic profile, and frequently this is a factor determining therapeutic success or failure. Since the first applications were performed until now, NGS studies on large patient cohorts have contributed to extend the information about the pathogenic basis of ALL. Today it is time to collect all the knowledge and select the most important targets to be analysed in a standardized clinical NGS workflow to establish precise international guidelines both for new diagnosis and for MRD monitoring. This effort will render NGS technology the real clinical instrument to make the much-vaunted precision medicine approach more feasible.

## Figures and Tables

**Table 1:** Recent Main studies concerning of next-generation sequencing (NGS) applications in B and T-acute lymphoblastic leukemia (ALL).

Reference	Cases Analyzed	NGS Approach	Main Findings
Safavi et al., 2015 [72]	11 hypodiploid B-ALL	WES, RNA-Seq	*IKZF3* and *FLT3* mutations in near-haploid (25–30 chr) cases; *TP53* mutations in low hypodiploidy (31–39 chr) and hypodiploid (40–4 chr) cases.
Paulsson et al., 2015 [71]	51 High hyperdiploid B-ALL	WGS and WES	Frequent involvement of the *RTK*-*RAS* pathway and of histone modifiers. No recurrent fusion gene–forming rearrangement found. The chromosomal gains were early events.
Fischer et al., 2015 [64]	TCF3-HLF−positive B-ALL	WGS, WES and RNA-Seq	Identification of recurrent intragenic deletions of *PAX5* or *VPREB1*, somatic mutations in the non-translocated allele of *TCF3* and a reduction of *PAX5* gene dosage in addition to *TCF3*-*HLF* fusion.
Andersson et al., 2015 [65]	67 ALL with MLL rearrangements (MLL-R)	WGS, WES, RNA-Seq, targeted NGS	Identification of activating mutations in kinase-*PI3K*-*RAS* signaling pathway components in 47% of cases; frequent mutations (45%) in epigenetic regulators in older children.
Messina et al., 2016 [69]	168 B-ALL lacking known fusion transcripts	WES and targeted NGS	Identification of 10.5 mutations and 9.1 CNAs /sample. The most frequently mutated pathways were *RAS*/*RTK* (26.8%) and *JAK*/*STAT* (12.5%) signaling.
Liu et al., 2016 [48]	203 B-ALL	WGS, RNA-Seq and Targeted deep sequencing	Identification of 29 new in-frame gene fusions and eight gene expression subgroups associated with characteristic genetic abnormalities: (*MEF2D* fusions, *TCF3*–*PBX1* fusions, *ETV6*–*RUNX1*–positive/*ETV6*–*RUNX1*–like, *DUX4* fusions, *ZNF384* fusions, *BCR*–*ABL1*/Ph–like, high hyperdiploidy, and *KMT2A* fusions).
Hu et al., 2017 [46]	30 T-ALL	WGS and RNA-Seq	Identification of 6.4 coding mutations per patient. Coding mutations were most frequently observed in *NOTCH1*, *FBXW7*, *USP7*, and *PTEN* genes. As regards noncoding mutations, three known T-ALL oncogenes, *LMO1*, *LMO2*, and *TAL1,* were those most frequently altered.
Gu et al., 2016 [50]	560 B-ALL	RNA-Seq	Identification of rearrangements between *MEF2D* (myocyte enhancer factor 2D) and five partner genes (*BCL9*, *CSF1R*, *DAZAP1*, *HNRNPUL1*, *SS18*). *MEF2D*-rearranged cases comprise 5.3% of ALL cases lacking recurring alterations and represent a distinct form of high-risk leukaemia.
Lilljebjorn et al., 2016 [49]	195 BCP-ALL	RNA-Seq	Identification of in-frame fusion genes in 65% of cases. Description of two new subtypes: 1) with *IGH-DUX4* or *ERG-DUX4* fusions, and DUX4 overexpression frequently co-occurring with intragenic *ERG* deletions (4% of cases); 2) with an*ETV6-RUNX1*-like gene-expression profile and coexisting *ETV6* and *IKZF1* alterations (3% of cases).
Zhang et al., 2016 [52]	1913 ALL	WGS, WES, RNA-Seq	Deregulation of *DUX4* and *ERG* genes in up to 7% of B-ALL. In all these cases a *DUX4* rearrangement and overexpression were present together with *ERG* transcriptional deregulation. *DUX4/ERG* ALL is associated with a favorable outcome.
Yasuda et al., 2016 [47]	73 Ph-neg AYA-ALL	RNA-Seq	Identification of *DUX4/IGH* fusion leading to a high level of expression of the DUX4 protein with an aberrant C terminus.
Quian et al., 2016 [63]	231 ALL	RNA-Seq	Identification of functional fusion genes in 54.1% of patients. Description of a distinct ALL subtype with *EP300*-*ZNF384* and *CREBBP*-*ZNF384* fusions causing epigenetic deregulation, offering potential for therapeutic targeting.
Li et al., 2018 [58]	1.223 BCP ALL	RNA-Seq	Identification of 14 gene expression subgroups (G1 to G14): eight previously described [48], and six additional gene expression subgroups: *PAX5* and *CRLF2* fusions; mutations in PAX5 (p.P80R); mutations in IKZF1 (p.N159Y), *IGH*–*CEBPE* fusion and mutations in ZEB2 (p.H1038R); *TCF3*/*4*–*HLF* fusion; *NUTM1* fusions. These molecular subgroups allow a better BCP ALL classification and prognostic stratification.
Vicente et al., 2015 [89]	155 T-ALL	Targeted NGS	*NOTCH1* and *CDKN2A*/*B* were altered in more than half of the cases, while an additional 37 genes were mutated in 4% to 20% of cases. The *IL7R-JAK* pathway was mutated in 27.7% of cases, suggesting a potential clinical application for JAK inhibitors in a significant proportion of patients with T-ALL.
Neumann et al., 2015 [90]	81 adult T-ALL	Targeted NGS	Recurrent mutations in *NOTCH1* (53%), *FBXW7* (10%), *WT1* (10%), *JAK3* (12%), *PHF6* (11%), and *BCL11B* (10%) in line with previous reports. Frequently affected pathways were the *JAK*/*STAT* pathway (18%) and epigenetic regulators (33%), both predominantly found in the unfavourable subgroup of early T-ALL. This could guide novel therapeutic approaches.
Feng et al., 2017 [91]	93 B-ALL, 28 T-ALL	Targeted NGS	About 90% of all cases harbored at least one mutation. The most frequently mutated genes were: *NOTCH1*, *JAK3*, *FBXW7*, *FAT1*, and *NRAS* (in T-ALL) and *FAT1*, *SF1*, *CRLF2*, *TET2*, and *PTPN1* (in B-ALL). B-ALL patients with the PTPN11 mutation and T-ALL patients with *NOTCH1* and/or *FBXW7* mutations showed better survival.
Liu et al., 2017 [51]	264 T-ALL	WGS, WES, RNA-Seq	Identification of 106 putative driver genes, half of which never previously seen in childhood T-ALL (e.g., *CCND3*, *CTCF*, *MYB*, *SMARCA4*, *ZFP36L2* and *MYCN*). Description of ten recurrently altered pathways, with associations between mutated genes and pathways, and the stage or subtype of T-ALL.
Chen et al., 2018 [92]	61 adult and 69 pediatric T-ALL cases	RNA-Seq	Identification of 36 gene fusion transcripts, *SET-NUP214* being highly related to adult cases. Identification of 18 new fusions (e.g., *ZBTB16-ABL1*, *TRA-SALL2*, and involvement of *NKX2-1)*. Up-regulation of *HOXA*, *MEF2C*, and *LYL1* often present in adult cases; *TAL1* overexpression mainly detected in the pediatric group.
Kimura et al., 2019 [104]	30 pediatric T-ALL	WES, targeted NGS	Identification of *NOTCH1*/*FBXW7* alterations in about 73% of cases at both diagnosis and relapse. Alterations in the heterodimerization domain were the most frequent (40.0%) at diagnosis, whereas PEST domain alterations were the most frequent at relapse (54.5%).

* Whole Genome Sequencing (WGS); Transcriptome sequencing (RNA-seq); Whole Exome Sequencing (WES) and Targeted gene sequencing (targeted NGS); adolescents and young adults (AYA).

## Abbreviations

ALL	acute lymphoblastic leukemia
NGS	next-generation sequencing
MRD	minimal residual disease
WGS	whole genome sequencing
RNA-seq	transcriptome sequencing
WES	whole exome sequencing
CNAs	copy number alterations
TKIs	tyrosine kinase inhibitors
ETP	early thymic progenitors
INDEL	insertions/deletions
SVs	structural variations
*MLL*	Mixed lineage leukemia
HT-NGS	High Throughput NGS
PCR	polymerase chain reaction
ASO	allele-specific oligonucleotide
RQ-PCR	quantitative PCR
SS	Sanger sequencing
